# Supplementary material for: Prescribed fire regimes influence responses of fungal and bacterial communities on new litter substrates in a brackish tidal marsh
Source: PLoS One. 2024 Oct 1;19(10):e0311230. doi: 10.1371/journal.pone.0311230 (PMC11444421; doi:10.1371/journal.pone.0311230)

Diagrams of the fire histories and experimental and plot design. Fire histories, shown on left, illustrate the date of each fire (red symbol) that occurred over the 10 years prior to the onset of the study in each fire regime (R). The field portion experiment, shown on right, takes place over 6 months from deployment on day 0 to the final collection date in December 2022. Plots and litter bags are classified by fire regime (R), litter load (L), sampling time (D) and collection date. Plots are unpaired and independent despite adjacent placement in table.

There were three studied fire regimes: R1, R4, and R5 which were burnt one, four, and five times, respectively, in the 10 years preceeding the study. The most recent fires prior to our study occurred 31 months (R1, December 2019), 15 months (R4, April 2021), and 20 months (R5, November 2020). 14 plots were established within each fire regime, for a total of 42 plots established in the study. Plots were sampled over a 6 month period for the current study. Each plot was assigned to receive one of two litter loads (L), L1 (1x litter load) or L2 (2x litter load), so litter loads are nested within each fire regime, with a total of 7 plots per each of 6 regime*load combinations. Within each plot, 4 litter bags were placed on day 0 of deployment in July 2022. Plots were then revisited after 60, 120, and 150 days (D) to collect litter bags to assess changes over time. These time points were designated D060, D120, and D150, respectively. Two bags were collected at D060, one bag was collected at D120, and the final bag was collected at D150. The below diagram shows the experimental design and sampling structure of plots. Plots are unpaired and independent despite adjacent placement in table.


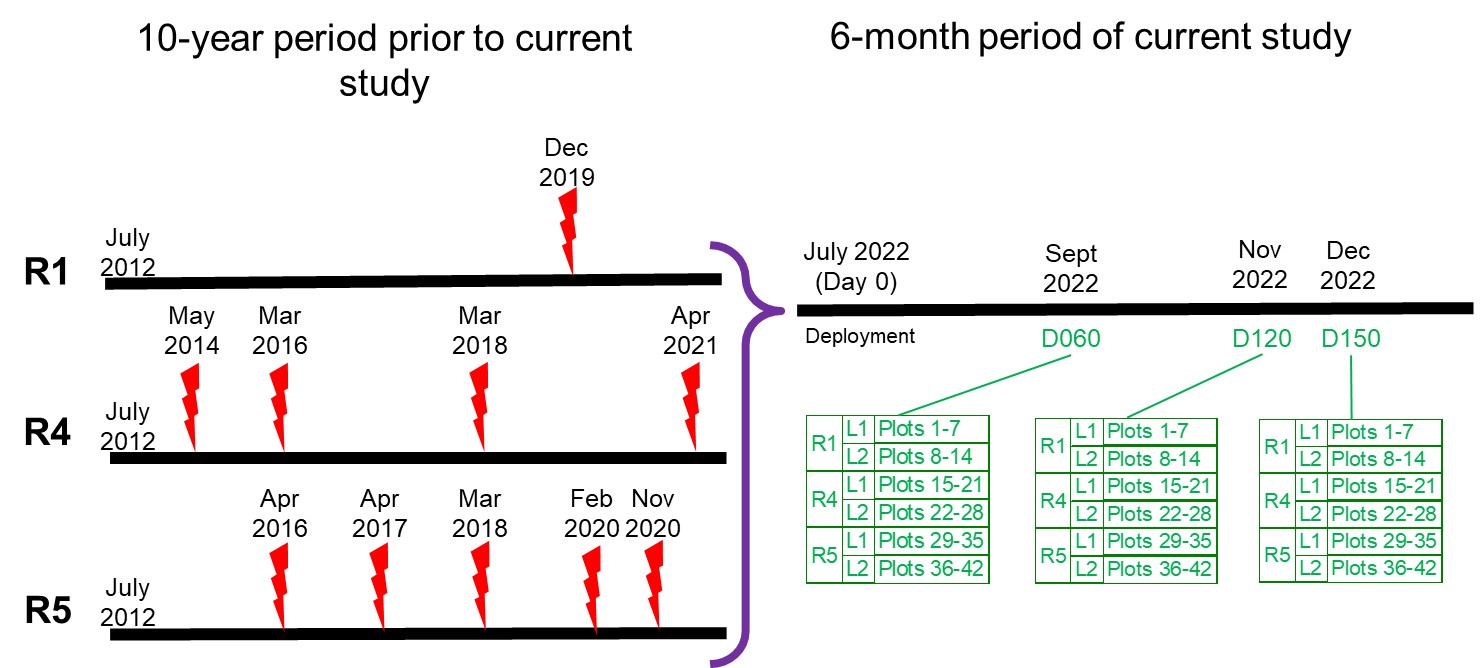

Supplement: S1 File — Fire histories, shown on left, illustrate the date of each fire (red symbol) that occurred over the 10 years prior to the onset of the study in each fire regime (R). The field portion experiment, shown on right, takes place over 6 months from deployment on day 0 to the final collection date in December 2022. Plots and litter bags are classified by fire regime (R), litter load (L), sampling time (D) and collection date. Plots are unpaired and independent despite adjacent placement in table. (DOCX) [file pone.0311230.s001.docx]
