# Supplementary material for: Prescribed fire regimes influence responses of fungal and bacterial communities on new litter substrates in a brackish tidal marsh
Source: PLoS One. 2024 Oct 1;19(10):e0311230. doi: 10.1371/journal.pone.0311230 (PMC11444421; doi:10.1371/journal.pone.0311230)
Supplement: S2 File — Permutational multivariate analysis of variance (PERMANOVA) summary table of treatment effects on the plant community composition measured at the onset of the study. Treatment effects were evaluated using Type I sums of squares on interactions and lower terms. Significance:. = 0.05<p<0.1, * = 0.01<p<0.05, ** = 0.001<p<0.01, *** = p<0.001. (DOCX) [file pone.0311230.s002.docx]

PERMANOVA summary table of treatment effects on the plant community composition. Plots were established in 3 fire regimes (R) of interest. Each plot was assigned to receive one of two litter loads (L). Within each plot, plant communities were identified and their relative abundances were used in a PERMANOVA to identify the effects of fire regime, litter load, and the fire regime*litter load interaction on the plant community composition. Output of the PERMANOVA includes degrees of freedom, sums of squares, R^2^ which indicates the percentage of plant community composition that was explained by that effect, a pseudo-F statistic, a p-value, and an indicator of significance. Significant effects indicate significant differences in plant community based on that effect. Treatment effects were evaluated using Type I sums of squares on interactions and lower terms. Significance: . = 0.05<p<0.1, * = 0.01<p<0.05, ** = 0.001<p<0.01, *** = p<0.001.

| Effect | Df | Sums of Squares | R^2^ | Pseudo-F | p-value | Significance |
| --- | --- | --- | --- | --- | --- | --- |
| Regime | 2 | 11.1205 | 0.44571 | 14.5057 | 0.001 | *** |
| Load | 1 | 0.2316 | 0.00928 | 0.6042 | 0.604 |  |
| Regime*Load | 2 | 0.5653 | 0.02266 | 0.7373 | 0.587 |  |
| Residual | 34 | 13.0327 | 0.52235 |  |  |  |
| Total | 39 | 24.9501 | 1 |  |  |  |
