# Supplementary material for: Prescribed fire regimes influence responses of fungal and bacterial communities on new litter substrates in a brackish tidal marsh
Source: PLoS One. 2024 Oct 1;19(10):e0311230. doi: 10.1371/journal.pone.0311230 (PMC11444421; doi:10.1371/journal.pone.0311230)
Supplement: S3 File — Contrasts were performed for significant main and interaction effects for each linear mixed model or Permutational multivariate analysis of variance (PERMANOVA) analysis, and were all compiled and presented here. Under each analysis heading are the significant main or interaction effects identified in the global test for that analysis, that describe the effect, the contrasts, and correction for significance. Under each significant effect are the null hypotheses (H0) for the relevant contrasts, and for each contrast, either a pseudo-F or a chi square statistic, the degrees of freedom for the test, the statistic, and the p-value. Contrasts were evaluated using Type III sums of squares with Bonferroni corrections applied to significance levels for each set of contrasts evaluated for each analysis. For contrasts involving fire regime, litter load, or their interaction, a significance level of α = 0.01 was used; for contrasts involving effects over time, α = 0.0033 was used to account for the multiple analyses. Red text indicates a significant contrast and difference. (DOCX) [file pone.0311230.s003.docx]

Contrasts were performed to analyze differences in various metrics by different treatment combinations of fire regime, litter load, their interaction, and changes in these effects over time. There were three studied fire regimes (R): R1, R4, and R5 which were burnt one, four, and five times, respectively, in the 10 years preceeding the study. Plots were established within each fire regime and assigned to receive one of two litter loads (L), L1 (1x litter load) or L2 (2x litter load). Litte bags were deployed in plots and retrieved after 60, 120, and 150 days (D), corresponding to time points D060, D120, and D150.

The experimental design resulted in up to 16 *a priori* null hypotheses regarding effects that could be tested as orthogonal contrasts in each statistical analysis. Each section includes the PERMANOVA or linear mixed model analysis performed on: plant community composition, litter bag mass change, alpha diversity metrics, and fungal and bacterial community composition. Under each analysis heading are the significant main or interaction effects identified in the global test for that analysis, that describe the effect, the contrasts, and correction for significance. Under each significant effect are the null hypotheses (H0) for the relevant contrasts, and for each contrast, either a pseudo-F or a chi square statistic, the degrees of freedom for the test, the statistic, and the p-value. Significant effects and their associated contrasts are presented here. Red text indicates a significant contrast and difference.

Two main effects [fire regime (R) and litter load (L)] result in one 2-way interaction (R x L), each with associated null hypotheses, all of which may or may not vary over time.

The effect of fire regime contained two orthogonal null hypotheses associated with comparisons of fire regimes, (R1 x R4 and R5) and (R4 x R5), designed to test the effect of low versus high frequency fire regimes, and the effect of 4 versus 5 fires in 10 years. The effect of litter load had two levels L1 and L2, and was only explored in contrasts if it was involved in interaction terms. The effect of repeated measures (time) contained two orthogonal null hypotheses associated with comparisons of sampling time, (D060 x D120 and D150) and (D120 and D150), designed to test variation in main effects when sampled at 60 days versus 120 and 150 days after plot establishment, and 120 days versus 150 days after plot establishment.

For each contrast made using a general linear mixed model, generalized linear mixed model, ANOVA, or PERMANOVA, the relevant statistic (F or Chi square value) and associated p-value are provided. Significant contrasts (i.e. rejection of null hypotheses) are indicated with red font.

For each analysis, a global test was performed using the 2-way interaction of fire regime*litter load over time, its lower main effects of fire regime and litter load, the plant community composition if appliable, and changes in each effect over time if appliable. Not all effects in each global test were significant; only significant main and interaction effects were evaluated post-hoc with contrasts. The contrasts for these significant effects are presented here. Type III sums of squares was used in each contrast, with Bonferroni corrections applied to each analysis’ set of contrasts.

1. Plant community composition
   1. Fire regime was a significant main effect, and had these 2 contrasts, with a Bonferroni correction of α = 0.05/5 = 0.01:
      1. H_0_: Plant community composition does not differ in R1 from R4 & R5. **F_(1, 36)_ = 27.847, p < 0.001.**
      2. H_0_: Plant community composition does not differ between R4 and R5. **F_(1,36)_ = 1.444, p = 0.218.**
2. Litter Bag Mass Change
   1. Fire regime was a significant main effect, and had these 2 contrasts, with a Bonferroni correction of α = 0.05/5 = 0.01:
      1. H_0_: Litter bag mass change does not differ in R1 from R4 & R5. **F_(1, 21)_ = 32.529, p < 0.001.**
      2. H_0_: Litter bag mass change does not differ between R4 and R5. **F_(1,21)_ = 1.390, p = 0.252.**
3. Fungal Community Composition
   1. Regime was a significant main effect, and had these 2 contrasts, with a Bonferroni correction of α = 0.05/5 = 0.01:
      1. H_0_: Fungal community composition does not differ in R1 from R4 & R5. **F_(1,29)_ = 16.2728, p < 0.001.**
      2. H_0_: Fungal community composition does not differ between R4 and R5. **F_(1,29)_ = 14.2556, p < 0.001.**
   2. Regimes varied by time, and had these 4 contrasts, with a Bonferroni correction of α = 0.01/3 = 0.0033:
      1. H_0_: Fungal community composition does not differ in R1 from R4 & R5 at time D060 compared to times D120 & D150. **F_(1,54)_ =** **2.8086, p < 0.001.**
      2. H_0_: Fungal community composition does not differ in R1 from R4 & R5 at time D120 compared to time D150. **F_(1,54)_ = 1.0369, p = 0.4768.**
      3. H_0_: Fungal community composition does not differ between R4 and R5 at time D060 compared to times D120 & D150. **F_(1,54)_ = 3.9194, p < 0.001.**
      4. H_0_: Fungal community composition does not differ between R4 and R5 at time D120 compared to time D150. **F_(1,54)_ = 1.0597, p = 0.3948.**
4. Bacterial Community Composition
   1. Regime was a significant main effect, and had these 2 contrasts, with a Bonferroni correction of α = 0.05/5 = 0.01:
      1. H_0_: Bacterial Community Composition does not differ in R1 from R4 & R5. F**_(1,29)_ =** **19.729, p < 0.001.**
      2. H_0_: Bacterial Community Composition does not differ between R4 and R5. F**_(1,29)_ =** **15.571, p < 0.001.**
   2. Regimes varied by time, and had these 4 contrasts, with a Bonferroni correction of α = 0.01/3 = 0.0033:
      1. H_0_: Bacterial community composition does not differ in R1 from R4 & R5 at time D060 compared to times D120 & D150. **F_(1,54)_ = 4.2744, p < 0.001.**
      2. H_0_: Bacterial community composition does not differ in R1 from R4 & R5 at time D120 compared to time D150. **F_(1,54)_ = 1.7700, p = 0.0273.**
      3. H_0_: Bacterial community composition does not differ between R4 and R5 at time D060 compared to times D120 & D150. **F_(1,54)_ = 3.7391, p < 0.001.**
      4. H_0_: Bacterial community composition does not differ between R4 and R5 at time D120 compared to time D150. **F_(1,54)_ = 2.1086, p = 0.0032.**
5. Alpha Diversity Metrics
   1. Fungal Species Richness
      1. Regime was a significant main effect, and had these 2 contrasts, with a Bonferroni correction of α = 0.05/5 = 0.01:
         1. H_0_: Fungal species richness does not differ in R1 from R4 & R5. **X^2^_(1)_ = 16.0011, p = 0.001.**
         2. H_0_: Fungal species richness does not differ between R4 and R5. **X^2^_(1)_ = 0.0050, p = 0.944.**
      2. Regimes varied by time, and had these 4 contrasts, with a Bonferroni correction of α = 0.01/3 = 0.0033:
         1. H_0_: Fungal species richness does not differ in R1 from R4 & R5 at D060 compared to D120 & D150. **X^2^_(1)_ = 25.6777, p < 0.001.**
         2. H_0_: Fungal species richness does not differ in R1 from R4 & R5 between D120 and D150. **X^2^_(1)_ = 0.848, p = 0.3571098.**
         3. H_0_: Fungal species richness does not differ between R4 and R5 at D060 compared to D120 & D150. **X^2^_(1)_ = 0.0053, p = 0.9420170.**
         4. H_0_: Fungal species richness does not differ between R4 and R5 between D120 and D150. **X^2^_(1)_ = 5.7721, p = 0.0162827.**
      3. Load varied by time, and had these 2 contrasts, with a Bonferroni correction of α = 0.01/3 = 0.0033:
         1. H_0_: Fungal species richness does not differ between L1 and L2 at D060 compared to D120 & D150. **X^2^_(1)_ = 10.1177, p = 0.001469.**
         2. H_0_: Fungal species richness does not differ between L1 and L2 between D120 and D150. **X^2^_(1)_ = 2.6227, p = 0.1053482.**
      4. Regime*Load was a significant interaction effect, and had these 2 contrasts, with a Bonferroni correction of α = 0.05/5 = 0.01:
         1. H_0_: Fungal species richness does not differ in R1 from R4 & R5 with different litter loads. **X^2^_(1)_ = 60.9725, p < 0.001.**
         2. H_0_: Fungal species richness does not differ between R4 and R5 with different litter loads. **X^2^_(1)_ = 10.9529, p < 0.001.**
      5. Regime*Load varied by time, and had these 4 contrasts, with a Bonferroni correction of α = 0.01/3 = 0.0033:
         1. H_0_: Fungal species richness does not differ in R1 from R4 & R5 with different litter loads at D060 compared to D120 & D150. **X^2^_(1)_ = 9.0995, p = 0.002557.**
         2. H_0_: Fungal species richness does not differ in R1 from R4 & R5 with different litter loads between D120 and D150. **X^2^_(1)_ = 1.2057, p = 0.2721821.**
         3. H_0_: Fungal species richness does not differ between R4 and R5 with different litter loads at D060 compared to D120 & D150. **X^2^_(1)_ = 1.2574, p = 0.2621411.**
         4. H_0_: Fungal species richness does not differ between R4 and R5 with different litter loads between D120 and D150. **X^2^_(1)_ = 5.0393, p = 0.0247786.**
   2. Fungal Evenness
      1. No effects were significant, so no contrasts were performed for this metric.
   3. Fungal Shannon Diversity
      1. No effects were significant, so no contrasts were performed for this metric
   4. Bacterial Species Richness
      1. Regime was a significant main effect, and had these 2 contrasts, with a Bonferroni correction of α = 0.05/5 = 0.01:
         1. H_0_: Bacterial species richness does not differ in R1 from R4 & R5. **X^2^_(1)_ = 0.0819, p = 0.774767.**
         2. H_0_: Bacterial species richness does not differ between R4 and R5. **X^2^_(1)_ = 200.0105, p < 0.001.**
      2. Regimes varied by time, and had these 4 contrasts, with a Bonferroni correction of α = 0.01/3 = 0.0033:
         1. H_0_: Bacterial species richness does not differ in R1 from R4 & R5 at D060 compared to D120 & D150. **X^2^_(1)_ = 79.6147, p < 0.001.**
         2. H_0_: Bacterial species richness does not differ in R1 from R4 & R5 between D120 and D150. **X^2^_(1)_ = 75.9241, p < 0.001.**
         3. H_0_: Bacterial species richness does not differ between R4 and R5 at D060 compared to D120 & D150. **X^2^_(1)_ = 0.0370, p = 0.8473811.**
         4. H_0_: Bacterial species richness does not differ between R4 and R5 between D120 and D150. **X^2^_(1)_ = 224.3326, p < 0.001.**
      3. Load varied by time, and had these 4 contrasts, with a Bonferroni correction of α = 0.01/3 = 0.0033:
         1. H_0_: Bacterial species richness does not differ between L1 and L2 at D060 compared to D120 & D150. **X^2^_(1)_ = 87.543, p < 0.001.**
         2. H_0_: Bacterial species richness does not differ between L1 and L2 between D120 and D150. **X^2^_(1)_ = 17.124, p < 0.001.**
      4. Regime*Load was a significant interaction effect, and had these 2 contrasts, with a Bonferroni correction of α = 0.05/5 = 0.01:
         1. H_0_: Bacterial species richness does not differ in R1 from R4 & R5 with different litter loads. **X^2^_(1)_ = 57.3062, p < 0.001.**
         2. H_0_: Bacterial species richness does not differ between R4 and R5 with different litter loads. **X^2^_(1)_ = 89.7222, p < 0.001.**
      5. Regime*Load varied by time, and had these 4 contrasts, with a Bonferroni correction of α = 0.01/3 = 0.0033:
         1. H_0_: Bacterial species richness does not differ in R1 from R4 & R5 with different litter loads at D060 compared to D120 & D150. **X^2^_(1)_ = 144.3423, p < 0.001.**
         2. H_0_: Bacterial species richness does not differ in R1 from R4 & R5 with different litter loads between D120 and D150. **X^2^_(1)_ = 20.4303, p < 0.001.**
         3. H_0_: Bacterial species richness does not differ between R4 and R5 with different litter loads at D060 compared to D120 & D150. **X^2^_(1)_ = 113.3033, p < 0.001.**
         4. H_0_: Bacterial species richness does not differ between R4 and R5 with different litter loads between D120 and D150. **X^2^_(1)_ = 71.4078, p < 0.001.**
   5. Bacterial Evenness
      1. Regime was a significant main effect, and had these 2 contrasts, with a Bonferroni correction of α = 0.05/5 = 0.01:
         1. H_0_: Bacterial evenness does not differ in R1 from R4 & R5. **X^2^_(1)_ = 1.2694, p =** **0.259879.**
         2. H_0_: Bacterial evenness does not differ between R4 and R5. **X^2^_(1)_ = 11.2465, p < 0.001.**
      2. Regime*Load was a significant interaction effect, and had these 2 contrasts, with a Bonferroni correction of α = 0.05/5 = 0.01:
         1. H_0_: Bacterial evenness does not differ in R1 from R4 & R5 with different litter loads. **X^2^_(1)_ =** **10.2770, p = 0.001347.**
         2. H_0_: Bacterial evenness does not differ between R4 and R5 with different litter loads. **X^2^_(1)_ =** **0.3284, p = 0.5666097.**
   6. Bacterial Shannon Diversity
      1. Regime was a significant main effect, and had these 2 contrasts, with a Bonferroni correction of α = 0.05/5 = 0.01:
         1. H_0_: Bacterial Shannon Diversity does not differ in R1 from R4 & R5. **X^2^_(1)_ = 0.0111, p =** **0.91590.**
         2. H_0_: Bacterial Shannon Diversity does not differ between R4 and R5. **X^2^_(1)_ = 71.7561, p < 0.001.**
      2. Regimes varied by time, and had these 4 contrasts, with a Bonferroni correction of α = 0.01/3 = 0.0033:
         1. H_0_: Bacterial Shannon diversity does not differ in R1 from R4 & R5 at time D060 compared to times D120 & D150. **X^2^_(1)_ = 1.3603, p = 0.24349.**
         2. H_0_: Bacterial Shannon diversity does not differ in R1 from R4 & R5 at time D120 compared to time D150. **X^2^_(1)_ = 3.1739, p = 0.07482.**
         3. H_0_: Bacterial Shannon diversity does not differ between R4 and R5 at time D060 compared to times D120 & D150. **X^2^_(1)_ = 15.593, p < 0.001.**
         4. H_0_: Bacterial Shannon diversity does not differ between R4 and R5 at time D120 compared to time D150. **X^2^_(1)_ = 19.107, p < 0.001.**
