# Supplementary material for: Prescribed fire regimes influence responses of fungal and bacterial communities on new litter substrates in a brackish tidal marsh
Source: PLoS One. 2024 Oct 1;19(10):e0311230. doi: 10.1371/journal.pone.0311230 (PMC11444421; doi:10.1371/journal.pone.0311230)
Supplement: S4 File — Table of summary statistics of litter bag mass gained per fire regime (R) and litter load (L) treatment at each sampled time point (D). Mean, 95% confidence interval, and lowest and highest mass gains were calculated across all plots within each treatment combination for the 131 total bags that were successfully retrieved. (DOCX) [file pone.0311230.s004.docx]

Summary table of the litter bag mass gained in each fire regime*litter load*time combination. There were 3 fire regimes of interest, R1, R4, and R5 corresponding to one, four, and five fires within the last 10 years preceeding the study. Each plot was assigned to receive one of two litter loads (L), L1 (1x litter load) or L2 (2x litter load). Within each plot, 4 litter bags were placed on day 0 of deployment in July 2022. Plots were then revisited after 60, 120, and 150 days (D) to collect litter bags to assess changes over time. These time points were designated D060, D120, and D150, respectively. Two bags were collected at D060, one bag was collected at D120, and the final bag was collected at D150.

This table presents multiple metrics analyzed on the masses gained by each litter bag within each fire regime*litter load*time combination. Provided are the average mass gained by each litter bag, the 95% confidence intervals for the mean estimate, the number of litter bags retrieved for that combination, and the lowest and highest amounts of mass gained over all bags within that combination. There were 131 total bags that were successfully retrieved over 3*2*3 = 18 fire regime*liter load*time combinations.

| Regime | Load | Time | Mean | 95% CI | n | Lowest Mass Gain | Highest Mass Gain |
| --- | --- | --- | --- | --- | --- | --- | --- |
| R1 | L1 | D060 | 3.359 | 0.105 | 10 | 3.078 | 3.654 |
| R1 | L1 | D120 | 3.325 | 0.285 | 7 | 2.758 | 3.713 |
| R1 | L1 | D150 | 3.434 | 0.372 | 7 | 2.744 | 4.305 |
| R1 | L2 | D060 | 3.159 | 0.237 | 8 | 2.663 | 3.727 |
| R1 | L2 | D120 | 3.041 | 0.283 | 9 | 2.341 | 3.652 |
| R1 | L2 | D150 | 2.516 | 0.214 | 5 | 2.126 | 2.786 |
| R4 | L1 | D060 | 3.060 | 0.416 | 12 | 2.604 | 5.284 |
| R4 | L1 | D120 | 2.945 | 0.245 | 6 | 2.590 | 3.276 |
| R4 | L1 | D150 | 2.846 | 0.130 | 6 | 2.563 | 3.032 |
| R4 | L2 | D060 | 2.069 | 0.178 | 9 | 1.653 | 2.413 |
| R4 | L2 | D120 | 1.888 | 0.347 | 6 | 1.335 | 2.352 |
| R4 | L2 | D150 | 1.891 | 0.486 | 5 | 1.309 | 2.757 |
| R5 | L1 | D060 | 2.733 | 0.139 | 10 | 2.395 | 3.218 |
| R5 | L1 | D120 | 2.736 | 0.193 | 7 | 2.460 | 3.096 |
| R5 | L1 | D150 | 2.497 | 0.147 | 5 | 2.315 | 2.726 |
| R5 | L2 | D060 | 1.894 | 0.202 | 9 | 1.467 | 2.450 |
| R5 | L2 | D120 | 1.727 | 0.317 | 5 | 1.179 | 2.077 |
| R5 | L2 | D150 | 1.577 | 0.378 | 5 | 0.903 | 2.040 |
