# Supplementary material for: Prescribed fire regimes influence responses of fungal and bacterial communities on new litter substrates in a brackish tidal marsh
Source: PLoS One. 2024 Oct 1;19(10):e0311230. doi: 10.1371/journal.pone.0311230 (PMC11444421; doi:10.1371/journal.pone.0311230)
Supplement: S5 File — Repeated measures Analysis of Variance (ANOVA) table for treatment effects on the mass gained in litter bags. Treatment effects were evaluated using Type III sums of squares on interactions and lower terms. Significance:. = 0.05<p<0.1, * = 0.01<p<0.05, ** = 0.001<p<0.01, *** = p<0.001. (DOCX) [file pone.0311230.s005.docx]

ANOVA table for the general linear mixed model that evaluated the effects of the fire regime*litter load*time combinations on mass gained in litter bags. Plots were established in 3 fire regimes of interest. Each plot received one of two litter loads. Litter bags were deployed in each plots. Plots were revisited after certain time points to collect litter bags to assess changes over time.

Litter bags showed increases in mass that may significantly differ by certain effects; this ANOVA table shows the significance of each effect on litter bag mass increases. Provided are the effect, degrees of freedom numerator and denominator, F-statistic, p-value, and significance level. A significant effect indicates that litter bag masses differed by that effect. This analysis used Type III sums of squares and a significance level of α = 0.05 to compare differences in litter bag mass across the regime*load*time interaction effect and all main and 2-way interaction effects. The linear model considered fire regime and litter load as main fixed effects, with plot ID considered as a random effect over which repeated measures were taken. Significance: . = 0.05<p<0.1, * = 0.01<p<0.05, ** = 0.001<p<0.01, *** = p<0.001.

| Effect | Df Numerator | Df Denominator | F-statistic | p-value | Significance |
| --- | --- | --- | --- | --- | --- |
| Regime | 1 | 22.118 | 38.623 | 2.89E-06 | *** |
| Load | 1 | 22.118 | 67.1847 | 3.78E-08 | *** |
| Time | 2 | 64 | 3.1355 | 0.05023 | . |
| Regime*Load | 1 | 22.118 | 2.8701 | 0.10428 |  |
| Regime*Time | 2 | 64 | 0.7485 | 0.47719 |  |
| Load*Time | 2 | 64 | 0.8716 | 0.42318 |  |
| Regime*Load*Time | 2 | 64 | 3.0838 | 0.05265 | . |
