# Supplementary material for: Prescribed fire regimes influence responses of fungal and bacterial communities on new litter substrates in a brackish tidal marsh
Source: PLoS One. 2024 Oct 1;19(10):e0311230. doi: 10.1371/journal.pone.0311230 (PMC11444421; doi:10.1371/journal.pone.0311230)

Bar plots of average mass gain in litter bags by each fire regime*litter load*time combination. There were 3 fire regimes (R) of interest, R1, R4, and R5 corresponding to one, four, and five fires within the last 10 years preceeding the study. Each plot was assigned to receive one of two litter loads (L), L1 (1x litter load) or L2 (2x litter load). Within each plot, litter bags were placed on day 0 of deployment. Plots were then revisited after 60, 120, and 150 days (D) to collect litter bags to assess changes over time. These time points were designated D060, D120, and D150, respectively. Upon collection, each bag was weighed for mass.

For each fire regime and litter load combination, and at each time point, the average mass gained with +/- 95% confidence intervals for each mean estimate were calculated and are shown below on left. Additionally for each fire regime and litter load, the average mass gained with +/- 95% confidence intervals and Tukey HSD pairwise significance letter groupings were calculated and are shown on right. Treatments with similar letters above bars are statistically similar.


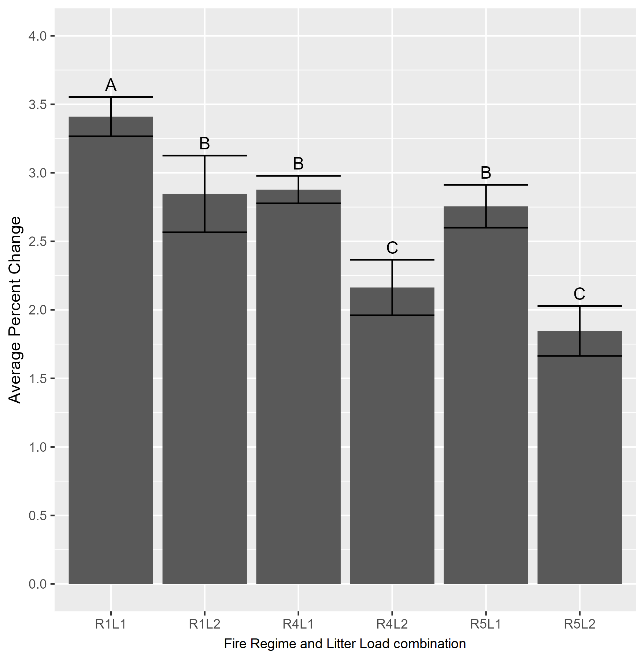

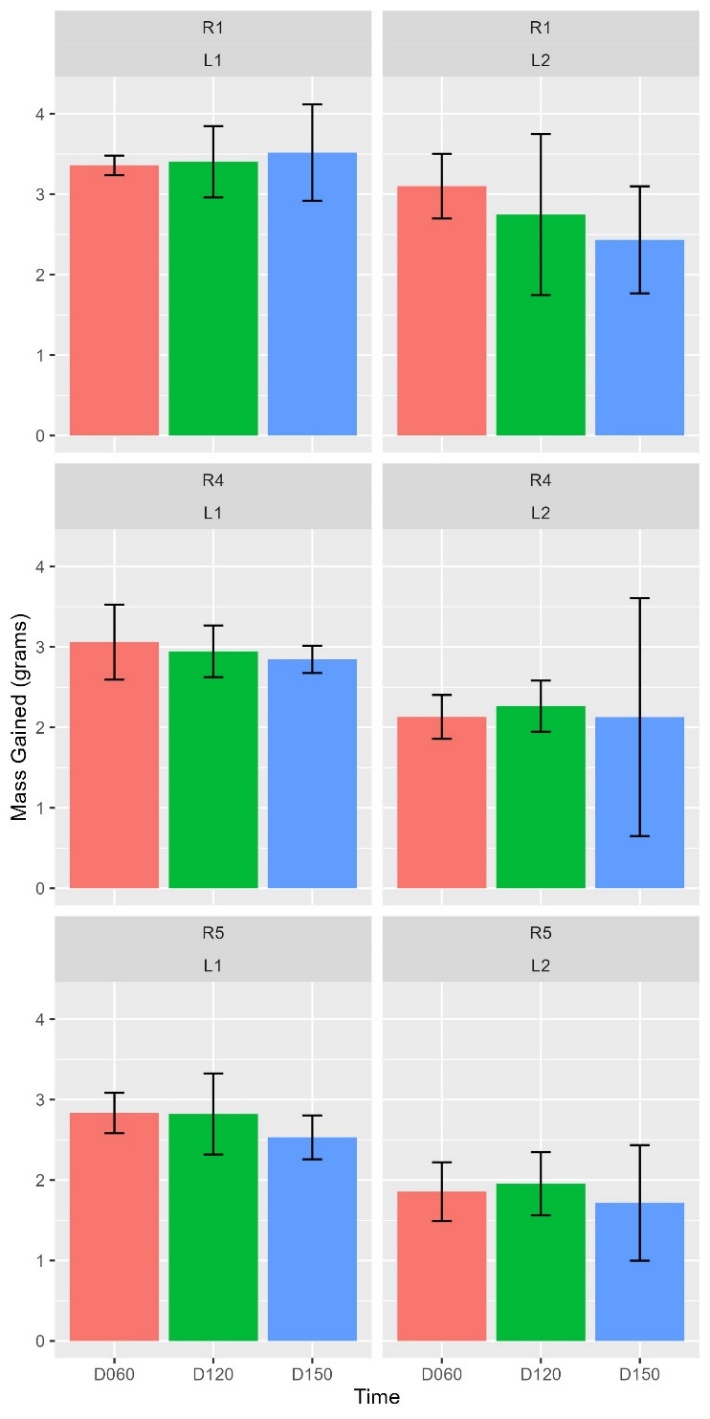

Supplement: S6 File — Average mass gain in litter bags (vertical bar), and +/- 95% confidence intervals (vertical lines) were calculated and are shown within each combination of fire regime (R) and litter load (L) treatments, at or over (left and right, respectively) the different sampling times (D). Tukey HSD pairwise comparison letter groupings were provided for the comparisons of fire regime and litter load. Treatments with similar letters above bars are statistically similar. (DOCX) [file pone.0311230.s006.docx]
