# Supplementary material for: Prescribed fire regimes influence responses of fungal and bacterial communities on new litter substrates in a brackish tidal marsh
Source: PLoS One. 2024 Oct 1;19(10):e0311230. doi: 10.1371/journal.pone.0311230 (PMC11444421; doi:10.1371/journal.pone.0311230)

Nonmetric Multi-Dimensional Scaling (NMDS) was used to compare microbial community compositions in the various treatments of fire regime and litter load, at sampled time points. There were three studied fire regimes (R): R1, R4, and R5 corresponding to one, four, and five fires in the 10 years preceeding the study. Plots were established within each fire regime. Each plot was assigned to receive one of two litter loads (L), L1 (1x litter load) or L2 (2x litter load). Within each plot, litter bags were placed on day 0 of deployment. Plots were then revisited after 60, 120, and 150 days (D) to collect litter bags to assess changes over time. These time points were designated D060, D120, and D150, respectively. DNA was extracted from these litter bag samples, sequencing data was processed into ESVs within each sample. ESV data was then transformed and distances were then calculated and then visualized using NMDS. The first two axes are shown, with each dot representing the community composition along those two NMDS axes. Each dot corresponds to a plot and community composition in that specific regime and time point combination. Plots and thus community composition can be assessed for patterns based on location in the NMDS ordination. More similar plots and community compositions cluster closer together. Overlaid geometric space shows spread and separation of plots of the same treatment combination.

This particular NMDS ordination of bacterial communities shows similarities of plots and bacterial community compositions within the separated fire regimes of R1 (A), R4 (B), and R5 (C) at different litter loads. Distances and ordination were based on bacterial ESV data in each treatment combination. Bacterial community compositions are shown here within each fire regime (R), and at different litter loads (L). Plots (symbols) are coded by litter load with symbol color and shape. Overlaid geometric space shows spread and separation of plots of the same litter load.


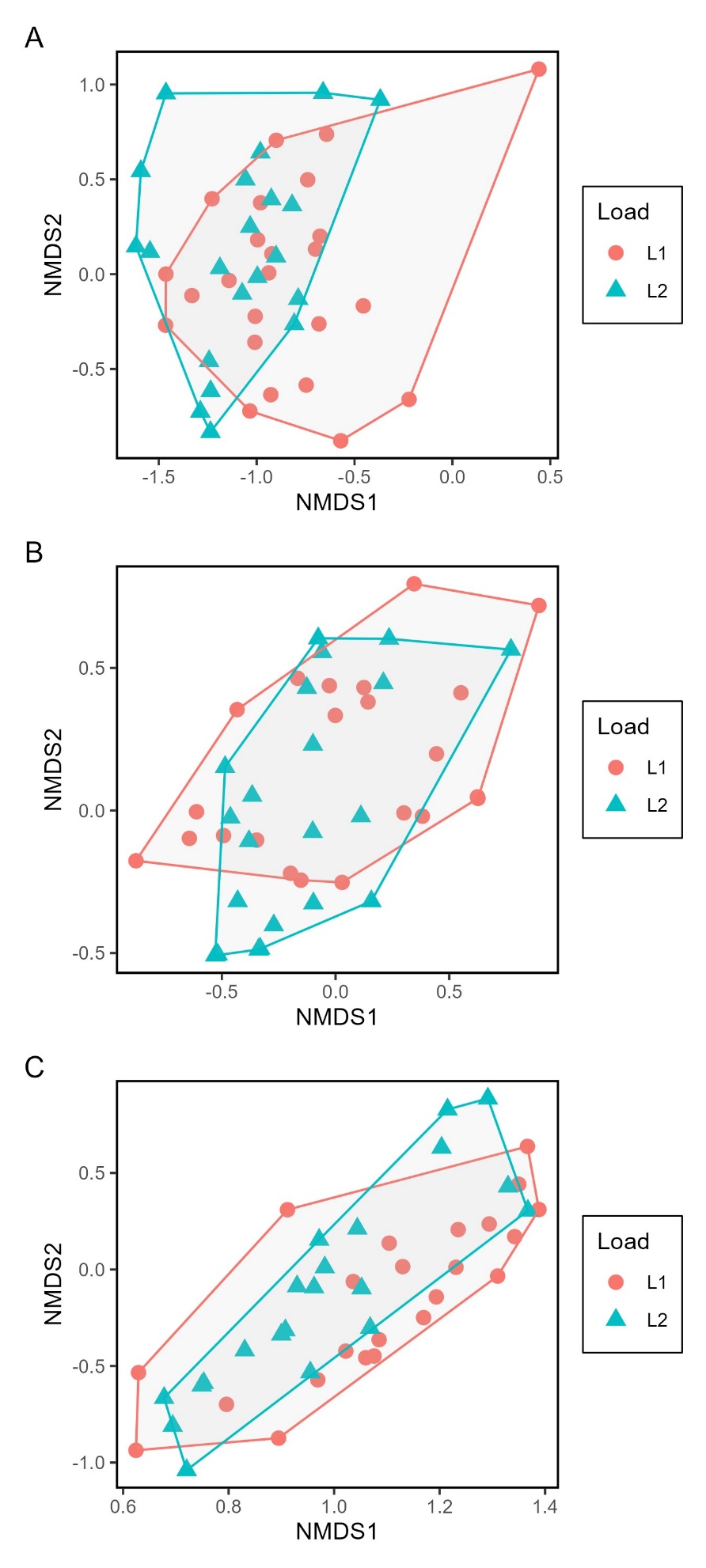

Supplement: S17 File — Non-metric, multi-dimensional scaling (NMDS) ordination of bacterial community compositions within the separated fire regimes of R1 (A), R4 (B), and R5 (C) at different litter loads. Distances and ordination were based on bacterial ESV data in each treatment combination. Bacterial community compositions are shown here within each fire regime (R), and at different litter loads (L). Plots (symbols) are coded by litter load with symbol color and shape. Overlaid geometric space shows spread and separation of plots of the same litter load. (DOCX) [file pone.0311230.s017.docx]
