# Supplementary material for: Prescribed fire regimes influence responses of fungal and bacterial communities on new litter substrates in a brackish tidal marsh
Source: PLoS One. 2024 Oct 1;19(10):e0311230. doi: 10.1371/journal.pone.0311230 (PMC11444421; doi:10.1371/journal.pone.0311230)
Supplement: S19 File — Permutational Multivariate Analysis of Variance (PERMANOVA) summary table of treatment effects and error terms on the fungal and bacterial community compositions based on the repeated measures design of the experiment. Treatment effects were evaluated using Type I sums of squares and a significance level of α = 0.01. Red font and bolding indicate significant effects with p<0.01. (DOCX) [file pone.0311230.s019.docx]

PERMANOVA summary table of treatment effects on the fungal and bacterial community composition. Plots were established in 3 fire regimes of interest. Each plot was assigned to receive one of two litter loads. Within each plot, plant communities were identified. Litter bags were deployed and collected after certain time points. DNA was extracted and processed into fungal and bacterial community ESV abundance data. The effects of fire regime, litter load, the fire regime*litter load interaction, dominant plant species, and interactions of each with time were analyzed on the fungal and bacterial community separately. Plots were treated as strata to restrict permutations in the PERMANOVA, thereby accounting for the variability associated with repeated measures. The PERMANOVA model included additional nested random effect terms that account for the non-independent effect of time on plots, with plots nested within regime in agreement with the experimental design. This allowed for more accurate decomposition of residual variation and degrees of freedom into within-plot and among-plot variation, as well as a mitigation of type I errors resultant from inflated residual error degrees of freedom. A significance level of α = 0.01 was used to account for the multiple analyses conducted on the microbial datasets. Output of the PERMANOVA includes degrees of freedom, sums of squares, R^2^ which indicates percentage of fungal or bacterial community composition that was explained by that effect, a pseudo-F statistic, and a p-value of significance. Significant effects are in red and indicate significant differences in fungal or bacterial community composition based on that effect. Treatment effects were evaluated using Type I sums of squares on interactions and lower terms.

|  | | Fungi | | | | Bacteria | | | |
| --- | --- | --- | --- | --- | --- | --- | --- | --- | --- |
| % Variation explained by all effects | | | | | 39.13% |  | | | 43.56% |
| % Variation explained by significant effects | | | | | 30.02% |  | | | 36.75% |
|  | Df | Sums of Squares | R^2^ | Pseudo-F | p-value | Sums of Squares | R^2^ | Pseudo-F | p-value |
| Regime | 2 | 103800 | 0.2199 | 10.9690 | **0.0001** | 120220 | 0.2614 | 15.5050 | **0.0001** |
| Load | 1 | 3022 | 0.0064 | 0.9988 | 0.3540 | 3818 | 0.0083 | 1.0829 | 0.2874 |
| Regime*Load | 2 | 7317 | 0.0155 | 1.1726 | 0.2057 | 8615 | 0.0187 | 1.2057 | 0.1461 |
| Juncus | 1 | 5802 | 0.0123 | 1.7322 | 0.0845 | 6973 | 0.0152 | 2.5739 | **0.0006** |
| Schoenoplectus | 1 | 4398 | 0.0093 | 1.4797 | 0.1577 | 6405 | 0.0139 | 2.6925 | **0.0013** |
| Spartina | 1 | 2722 | 0.0058 | 0.7702 | 0.6345 | 3281 | 0.0071 | 1.1432 | 0.2799 |
| Typha | 1 | 5878 | 0.0125 | 1.9631 | 0.0662 | 3046 | 0.0066 | 1.2700 | 0.2163 |
| Residual Among Plots | 29 | 98765 | 0.2092 |  |  | 89788 | 0.1952 |  |  |
|  |  |  |  |  |  |  |  |  |  |
| Time | 2 | 18270 | 0.0387 | 3.2851 | **0.0001** | 14401 | 0.0313 | 2.7415 | **0.0001** |
| Regime*Time | 4 | 19644 | 0.0416 | 1.8327 | **0.0002** | 21020 | 0.0457 | 2.0384 | **0.0001** |
| Load*Time | 2 | 4201 | 0.0089 | 0.9629 | 0.2966 | 4280 | 0.0093 | 0.9222 | 0.3239 |
| Regime*Load*Time | 4 | 9650 | 0.0204 | 1.0761 | 0.1232 | 8276 | 0.0180 | 0.8952 | 0.3309 |
| Residual Within Plots | 54 | 121017 | 0.2564 |  |  | 117340 | 0.2551 |  |  |
| Residual | 30 | 67520 | 0.1430 |  |  | 52451 | 0.1140 |  |  |
| Total | 131 | 472005 |  |  |  | 459914 |  |  |  |
