# Supplementary material for: Prescribed fire regimes influence responses of fungal and bacterial communities on new litter substrates in a brackish tidal marsh
Source: PLoS One. 2024 Oct 1;19(10):e0311230. doi: 10.1371/journal.pone.0311230 (PMC11444421; doi:10.1371/journal.pone.0311230)
Supplement: S20 File — Compiled Analysis of Variance (ANOVA) summary tables for the generalized linear mixed models built to evaluate treatment effects on fungal and bacterial richness, evenness, and Shannon diversity. Treatment effects were evaluated using Type III sums of squares, with a significance level of α = 0.01. Red font and bolding indicate p<0.01. (DOCX) [file pone.0311230.s020.docx]

ANOVA table for the generalized linear mixed models that were fit to evaluate treatment effects on different alpha diversity metrics of richness, evenness, and Shannon diversity for fungi and bacteria separately. Plots were established in 3 fire regimes of interest. Each plot was assigned to receive one of two litter loads. Litter bags were deployed and collected after certain time points. Within each plot, plant communities were identified. DNA was extracted and processed into fungal and bacterial community ESV abundance data. The effects of fire regime, litter load, the fire regime*litter load interaction, dominant plant species, and interactions of each with time were analyzed on alpha diversity metrics. For each metric, a generalized linear mixed model was fit using type III sums of squares with a significance level of α = 0.01 to assess effects of fire regime, litter load, their interaction, and their interactions with time. Species richness was modeled with a Poisson distribution and log link, species evenness with a beta distribution and logit link, and Shannon diversity with a gamma distribution and log link. Each linear model considered the fire regime*load*time interaction, with plot nested within fire regime as a random effect. An ANOVA was run on each generalized linear mixed model to assess the significance of each effect on the alpha diversity metric.

Below is the output ANOVA table of each analysis, including the effect, degrees of freedom numerator and denominator, F-statistic, p-value, and significance level. This ANOVA used Type III sums of squares and a significance level of α = 0.01. A significant effect indicates significant differences in that alpha diversity metric based on the significant effect. Red and bolded font indicates significant effects.

| Fungal Richness | X^2^ | Df | p-value |  | Bacterial Richness | X^2^ | Df | p-value |
| --- | --- | --- | --- | --- | --- | --- | --- | --- |
| Regime | 15.744 | 2 | **<0.001** |  | Regime | 220.299 | 2 | **<0.001** |
| Load | 55.65 | 1 | **<0.001** |  | Load | 37.770 | 1 | **<0.001** |
| Time | 82.851 | 2 | **<0.001** |  | Time | 24.482 | 2 | **<0.001** |
| Regime*Load | 70.936 | 2 | **<0.001** |  | Regime*Load | 135.068 | 2 | **<0.001** |
| Regime*Time | 29.834 | 4 | **<0.001** |  | Regime*Time | 1159.143 | 4 | **<0.001** |
| Load*Time | 13.436 | 2 | **0.0012** |  | Load*Time | 38.163 | 2 | **<0.001** |
| Regime*Load*Time | 25.607 | 4 | **<0.001** |  | Regime*Load*Time | 89.268 | 4 | **<0.001** |
|  | | | | | | | | |
| Fungal Evenness | X^2^ | Df | p-value |  | Bacterial Evenness | X^2^ | Df | p-value |
| Regime | 0.383 | 2 | 0.826 |  | Regime | 14.048 | 2 | **<0.001** |
| Load | 3.722 | 1 | 0.054 |  | Load | 10.387 | 1 | **0.0013** |
| Time | 0.774 | 2 | 0.679 |  | Time | 2.064 | 2 | 0.356 |
| Regime*Load | 2.705 | 2 | 0.259 |  | Regime*Load | 9.272 | 2 | **0.0097** |
| Regime*Time | 1.984 | 4 | 0.739 |  | Regime*Time | 1.961 | 4 | 0.743 |
| Load*Time | 1.758 | 2 | 0.415 |  | Load*Time | 0.389 | 2 | 0.823 |
| Regime*Load*Time | 1.428 | 4 | 0.839 |  | Regime*Load*Time | 1.124 | 4 | 0.891 |
|  | | | | | | | | |
| Fungal Shannon Diversity | X^2^ | Df | p-value |  | Bacterial Shannon Diversity | X^2^ | Df | p-value |
| Regime | 1.031 | 2 | 0.597 |  | Regime | 77.954 | 2 | **<0.001** |
| Load | 0.355 | 1 | 0.552 |  | Load | 0.014 | 1 | 0.906 |
| Time | 2.005 | 2 | 0.367 |  | Time | 1.468 | 2 | 0.480 |
| Regime*Load | 0.027 | 2 | 0.987 |  | Regime*Load | 2.743 | 2 | 0.254 |
| Regime*Time | 1.071 | 4 | 0.899 |  | Regime*Time | 38.095 | 4 | **<0.001** |
| Load*Time | 0.151 | 2 | 0.927 |  | Load*Time | 0.767 | 2 | 0.681 |
| Regime*Load*Time | 0.147 | 4 | 0.997 |  | Regime*Load*Time | 2.430 | 4 | 0.657 |
