# Supplementary material for: Prescribed fire regimes influence responses of fungal and bacterial communities on new litter substrates in a brackish tidal marsh
Source: PLoS One. 2024 Oct 1;19(10):e0311230. doi: 10.1371/journal.pone.0311230 (PMC11444421; doi:10.1371/journal.pone.0311230)

Boxplots for each alpha diversity metric for fungi and bacteria in fire regime*time combinations. There were three studied fire regimes (R): R1, R4, and R5 corresponding to one, four, and five fires in the 10 years preceeding the study. Plots were established within each fire regime. Each plot was assigned to receive one of two litter loads (L), L1 (1x litter load) or L2 (2x litter load). Within each plot, litter bags were placed on day 0 of deployment. Plots were then revisited after 60, 120, and 150 days (D) to collect litter bags to assess changes over time. These time points were designated D060, D120, and D150, respectively. DNA was extracted from these litter bag samples, sequencing data was processed into ESVs within each sample, and alpha diversity metrics were then calculated on the ESV data for fungi and bacteria separately.

Alpha diversity metrics of richness, evenness, and Shannon diversity were calculated separately for fungi and bacteria, and their distributions were visualized in boxplots. These boxplots show alpha diversity metrics within different fire regimes and litter loads at different sampling times, for fungal species richness (A), fungal evenness (B), fungal Shannon diversity (C); and bacterial species richness (D), bacterial evenness (E), and bacterial Shannon diversity (F). Boxplots show the alpha diversity metric in each fire regime (R), litter load (L), and sampling time (D) combination. Colors group similar time points across different fire regimes.


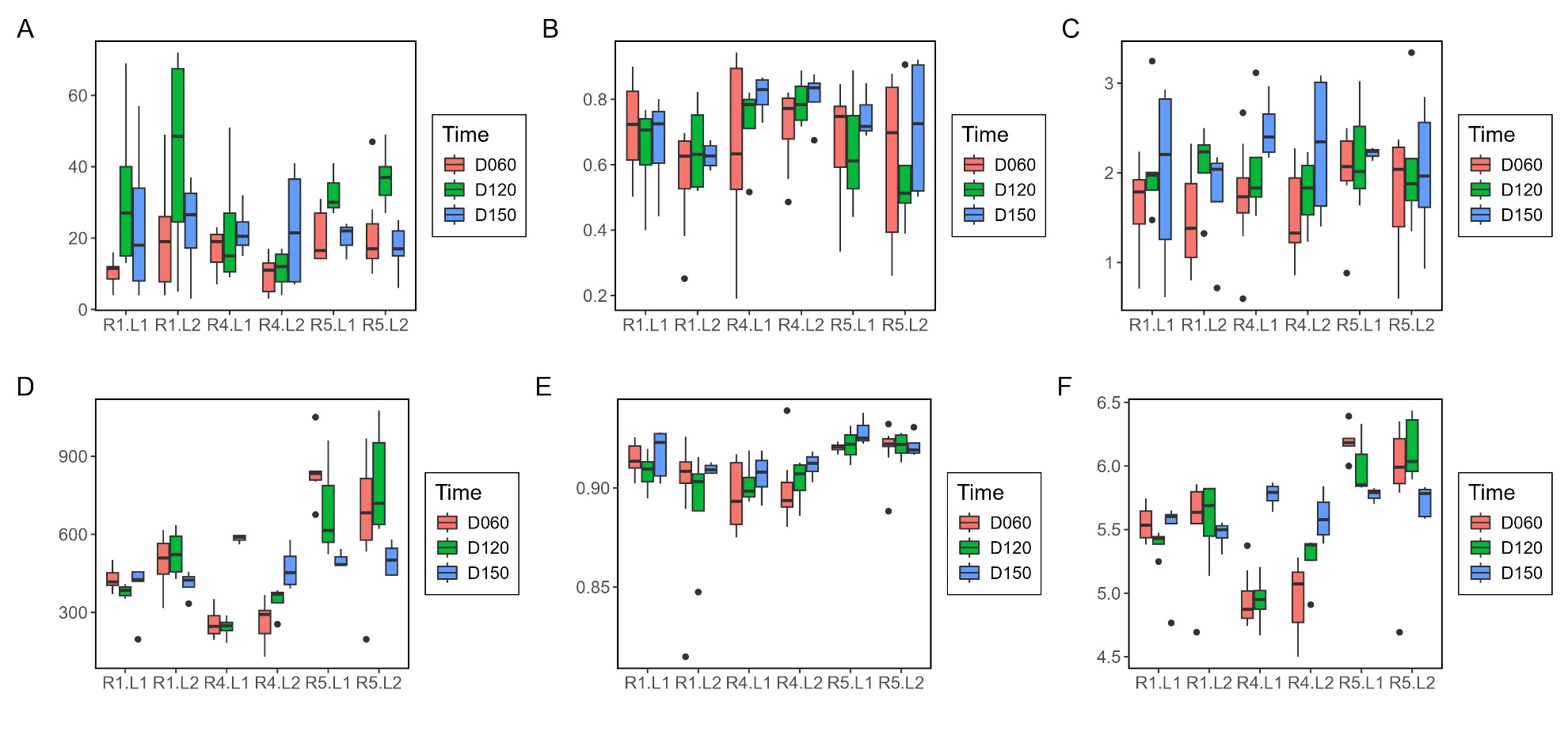

Supplement: S22 File — Boxplots showing alpha diversity metrics within different fire regimes and litter loads at different sampling times, for fungal species richness (A), fungal evenness (B), fungal Shannon diversity (C); and bacterial species richness (D), bacterial evenness (E), and bacterial Shannon diversity (F). Boxplots show the alpha diversity metric in each fire regime (R), litter load (L), and sampling time (D) combination. Colors group similar time points across different fire regimes. (DOCX) [file pone.0311230.s022.docx]
