# Supplementary material for: Prescribed fire regimes influence responses of fungal and bacterial communities on new litter substrates in a brackish tidal marsh
Source: PLoS One. 2024 Oct 1;19(10):e0311230. doi: 10.1371/journal.pone.0311230 (PMC11444421; doi:10.1371/journal.pone.0311230)
Supplement: Attachment — Submitted filename: Response to Reviewers.docx [file pone.0311230.s025.docx]

- **Editors and reviewers recommended reduction of the manuscript’s size and making it more concise and readable.**
  - Thank you for the suggestion. We were also concerned for the manuscript’s size when we first submitted it, so with your requests we’ve made great effort toward making the manuscript much better overall.
  - Aggressive reductions were implemented that targeted accessory information not central to the main story. One reviewer commented on the clear messages of the narrative we present, and those reductions were performed to maintain those clear messages and remove accessory information that was unnecessary for those messages.
  - We believe that the edits, although extensive, make the manuscript quite readable now, and hope that you find it much more pleasant to read.
- **Editors and reviewers recommended reducing methods considerably.**
  - Thank you for this suggestion as well. We were considering the methods also being very long, but thorough. One reviewer remarked on this too, stating that the “Material and Methods section is extensively well explained with all the sub-sections indicated very detailed.”
  - The original intention of the long methods, and the reason we kept it long for the first submission, was to explain the methods and their interpretation so the methods and results can be clearly understood and interpreted by everyone to which PLOS One caters.
    - We specifically wrote the methods with great detail so anyone with any level of familiarity with the methods could still understand the outputs, the results, and the figures and interpretations. Specifically, the methods were originally written in such a way that people with no familiarity with the analyses could still understand the analyses if they have foundational statistical knowledge.
    - Much information in the methods was dedicated to that purpose, but was ultimately more accessory to the purpose of the methods section, which is generally focused on describing the experiment to facilitate replication. Thus, much of this additional and accessory information was removed, so the methods currently retain all information needed for full experiment replication, but as a tradeoff, now require a level of familiarity with the analyses in order to interpret and understand.
    - As a compromise, information on interpreting the analyses was moved into supplemental files relevant to each analysis. This results in the main manuscript presenting highly concise methods that facilitate experimental replication, meanwhile the supplements present accessory information that facilitate results/data/analyses interpretations by any reader, including those with no familiarity with the analysis.
  - Methods have been reduced from 3382 words with headings, to 2680 words with headings. This is a 21% reduction.
    - Much of the removed content was ultimately that accessory information that was not directly relevant to the core of the research presented, but still helps readers of any familiarity understand the methods and analyses.
    - Much of this information was moved to the supplementary files should any reader wish to learn more about the method, or facilitate better interpretation of the data and results.
- **Editors and reviewers recommended merging results and discussion.**
  - Thank you all for the suggestion, and we understand the reasoning behind this suggestion, especially given the size of the manuscript originally submitted.
    - The narrative structure relies heavily on subsections that are each addressed in order, in both the results and discussion, so with the huge size, those subsections can get crossed when reading, and information become distorted.
  - The current version of the manuscript removed a lot of accessory information in both the results and discussion that together make the sections much more readable such that the presented information is much less likely to be crossed or confused. These sections are both much smaller due to extensive reworking of the results and discussion, so we believe that the narrative overall now benefits from keeping the sections separated.
    - We removed accessory content that was designed to help interpret the results. This content was, like the accessory information in the methods, ultimately put in the supplements since they were not needed for the main narrative, and were mainly designed to assist a reader of any familiarity to understand the results and data.
    - We also removed content in the results that presented nonsignificant results. This was done first because the manuscript’s overall size quickly grew due to the number of analyses performed and the number of these nonsignificant results that were presented. Second, these nonsignificant results crowded out and made less clear the significant results that were more central to our narrative.
    - We also removed content in the discussion that was simply repeating the results. This content was originally included for the reader’s convenience so they would not have to refer back to the results while reading the discussion. As a compromise, we rewrote many sentences of the discussion to indirectly remind the reader of the results. This framing then facilitated commentary on, and interpretation, of the results that would be expected for a discussion section.
    - We also removed content in the discussion that was ultimately not central to our main narrative, or were excessively detailed explanations of tangential topics. This results in the discussion having less meandering discussion, and instead having much more focused, plausible, and literature-supported explanations of our results.
  - The results section was reduced from 2817 words with headings, to 1669 words with headings. This is a 41% reduction.
  - The discussion section was reduced from 4089 words with headings, to 2462 words with headings. This is a 40% reduction.
  - Ultimately, we believe that these reductions together make the manuscript’s results and discussion sections much easier to read. The subsections help keep together the parts of the narrative, and in tandem with the far reduced size and the much improved readability, we believe these changes justify keeping the sections separate.
- **Editor requests meeting stylistic requirements for file naming**
  - Many thanks for this request. The author guidelines were not fully clear on file naming, especially for supplementary files, so your direction helps a lot with unifying the overall style.
  - All file naming styles and formats were corrected. In line citations were made stylistically similar, e.g. “Fig 1” and “S4 File.” Similarly, all file names were stylistically similar, e.g. “Fig1.tiff” and “S4_File.docx”
- **Editor requires full ethics statement be provided in the methods, with IRB or ethics committee mentioned.**
  - Thank you for the request. We were not fully sure about what was needed to include since the author guidelines were little ambiguous, so this request gives clear direction.
  - We did not use an IRB or ethics committee since our research and studied ecosystem does not involve oversight of these types of committees. However, at the very end of the methods, we added additional information regarding permitting and permission from the ecosystem managers, and information regarding management, and samples collected. We believe this transparency meets the requirements.
- **Editor requires addressing “data not shown” and data sharing requirements.**
  - Thank you for the request.
  - The problematic section was focused on showing the variation present in the inferred functions that was not shown by the functions we presented. This variation and this section were ultimately accessory information for the results, and were not a core part of the research being presented. This section was therefore removed and there is no longer any usage of “data not shown.”
- **Editor requires addressing copyrighted Google satellite imagery by either getting written permission or removing/replacing the image.**
  - Thank you for this request. We were especially unsure about usage of Google Satellite imagery, and their copyright policy and fair use guidelines were unclear in the context of our manuscript.
  - The problematic image was also accessory information presented in the methods, designed to show the spatial variability of the marsh. We unfortunately do not have any replacement photos, but we provide enough information in the methods for a reader to explore on their own the marsh and its variability. The problematic image and supplement was entirely removed since it was not ultimately necessary to the research presented. There currently remains no copyrighted imagery.
